# Hematopoietic Lineage Transcriptome Stability and Representation in PAXgene^™^ Collected Peripheral Blood Utilising SPIA Single-Stranded cDNA Probes for Microarray

**DOI:** 10.4137/bmi.s938

**Published:** 2008-08-25

**Authors:** Laura Kennedy, J. Keith Vass, D. Ross Haggart, Steve Moore, Michael E. Burczynski, Dan Crowther, Gino Miele

**Affiliations:** 1 Translational Medicine Research Collaboration Laboratory, Sir James Black Centre, University of Dundee, Dundee, DD1 5EH, U.K; 2 Biomarker Laboratory, Wyeth Research, Collegeville, U.S.A

**Keywords:** biomarkers, whole blood, globin reduction, paxgene^™^, NuGEN^™^

## Abstract

Peripheral blood as a surrogate tissue for transcriptome profiling holds great promise for the discovery of diagnostic and prognostic disease biomarkers, particularly when target tissues of disease are not readily available. To maximize the reliability of gene expression data generated from clinical blood samples, both the sample collection and the microarray probe generation methods should be optimized to provide stabilized, reproducible and representative gene expression profiles faithfully representing the transcriptional profiles of the constituent blood cell types present in the circulation. Given the increasing innovation in this field in recent years, we investigated a combination of methodological advances in both RNA stabilisation and microarray probe generation with the goal of achieving robust, reliable and representative transcriptional profiles from whole blood. To assess the whole blood profiles, the transcriptomes of purified blood cell types were measured and compared with the global transcriptomes measured in whole blood. The results demonstrate that a combination of PAXgene^™^ RNA stabilising technology and single-stranded cDNA probe generation afforded by the NuGEN Ovation RNA amplification system V2^™^ enables an approach that yields faithful representation of specific hematopoietic cell lineage transcriptomes in whole blood without the necessity for prior sample fractionation, cell enrichment or globin reduction. Storage stability assessments of the PAXgene^™^ blood samples also advocate a short, fixed room temperature storage time for all PAXgene^™^ blood samples collected for the purposes of global transcriptional profiling in clinical studies.

## Introduction

Development of high-quality biomarkers for disease progression, target engagement, pharmacodynamic effects and compound efficacy is central to translational research efforts aimed at establishing personalised medicine. However, identification of such biomarkers is frequently hampered by lack of access to target tissues with direct biological involvement. As such, peripheral blood (PB) has become an increasingly attractive source of surrogate material in place of target tissue as a focus for biomarker discovery and application, representing one of the most widely available and practical sources of biological material collected within the clinic and permitting a largely non-invasive method of repeated analyses within the same patient (Burczynski and Dorner, 2006).

In diseases such as autoimmune disorders and leukaemias, where PB lineages are directly involved in the disease process, disease-associated transcriptional profiles have been well established (Alizadeh et al. 2000; Baechler et al. 2003; Bullinger et al. 2004; Crow and Wohlgemuth, 2003; Golub et al. 1999; Han et al. 2003; Maas et al. 2002; McLean et al. 2004; Moos et al. 2002; Nowicki et al. 2003; Valk et al. 2004; Bennett et al. 2003). In recent years interest has also turned to the potential of PB as a surrogate tissue for the discovery of additional types of biomarkers in other scenarios. Given the highly dynamic cellular nature of the blood and its circulation throughout the body, it is likely that systemic responses to a disease state can be captured within the PB transcriptome. Several examples of successful biomarker discovery in transcriptome data derived from PB to date have been reported from pilot studies in neurological disorders (Borovecki et al. 2005; Hershey et al. 2004; Tang et al. 2005; Moore et al. 2005), oncology settings (Twine et al. 2003; Burczynski et al. 2005), SARS (Yu et al. 2005) and gastrointestinal disorders (Burczynski et al. 2006). This accumulating body of evidence supports the utility of peripheral blood transcriptional profiling for identification of biomarkers that may function as biomarkers of disease, evidence of pharmacodynamic effect, or even predictors of clinical outcomes and risk of toxicity. Whilst peripheral blood transcriptional profiling appears to hold great promise, there are several limitations to use of this matrix for gene expression studies that should be carefully considered in any study for resulting disease of pharmacodynamic biomarkers to hold any clinical utility (Mohr and Liew, 2007).

Transcriptional profiling from PB can be achieved using a number of methodologies and associated workflows; for example a) whole blood, b) enriched mononuclear fractions (PBMCs) or c) selected/enriched specific hematopoietic lineages. Naturally, each of these approaches possess distinct advantages and disadvantages. Hematopoietic lineage representation in PBMCs or selected/enriched fractions, whilst not requiring introduction of globin reduction strategies prior to microarray experiments, is naturally restricted to the lineages under study and several cell lineages are consequently omitted from further analysis. Global profiling of whole blood RNA, although preferable in order to capture the blood transcriptome in its entirety, has traditionally required globin reduction strategies prior to probe generation for microarray analysis in order to reduce the interference of globin transcripts with array probes. In addition to increasing processing steps, these procedures add the risk of artefactual modulation of the transcriptome and the utility of globin reduction strategies have been questioned in several reports (Liu et al. 2006; Li et al. 2008; Dumeaux et al. 2008).

The consistency of gene expression profiling data of whole blood is highly dependant on the sample stabilisation method used at phlebotomy or during storage (Debey et al. 2006; Rainen et al. 2002). Technologies for whole blood sample collection and storage exist, designed in an attempt to overcome problems associated with clinical collection and PB transcriptome stability. One of these, the PAXgene^™^ Blood RNA system (PreAnalytiX, Hornbrechtikon, Switzerland) consists of an evacuated PAXgene^™^ RNA tube for blood collection and a processing kit for isolation of total RNA from whole blood. The PAXgene^™^ collection tube contains a proprietary reagent that is reported to immediately stabilise intracellular RNA for up to 3 days at room temperature. The potential to minimize the requirement for urgent sample processing by increasing the clinical sample transcriptome stability in this matrix is central to the discovery of robust biomarkers.

Several groups have demonstrated the impact of PAXgene^™^ blood collection on RNA transcript stabilisation using quantitative PCR (qPCR) analyses. These studies have demonstrated the utility of the PAXgene^™^ system in restricting both initial and longer term (several days) *ex vivo* gene expression changes occurring after phlebotomy compared to conventional anticoagulant methods for blood collection (Muller et al. 2002; Pahl and Brune, 2002; Rainen et al. 2002; Thorn et al. 2005). The impact of different PAXgene^™^ storage protocols on RNA quantity and quality has also been investigated with several reports obtaining high quality RNA samples over a range (2 hrs, 9 hrs, 24 hrs and 5 days) of storage times at room temperature (Chai et al. 2005; Thach et al. 2003; Wang et al. 2004). When generating gene expression data using qPCR of selected transcripts as the biological readout, good comparisons between replicate samples has been shown, although variability between samples increases with longer incubation periods (Wang et al. 2004). Gene expression levels of a limited number of transcripts in PAXgene^™^-collected whole blood following 5 days room temperature storage have shown no alteration (Chai et al. 2005) compared to 24 hour storage. Conversely, a reduction in RNA integrity after storage in PAXgene^™^ at room temperature from 1 to 7 days has been reported and, even without apparent reduction in RNA integrity, specific transcript instability has been reported even with storage at +4 °C rather than room temperature (Kagedal et al. 2005). Considering the potential clinical impact of obtaining high quality and faithful gene expression profiling from peripheral blood, it is crucial to establish a consistent, robust and practical sample collection method for clinical blood samples.

Due to the high level of reticulocytes in peripheral blood there is a predominance of globin mRNA transcripts with the potential to (a) result in under-representation of non-globin transcripts and (b) impact on microarray data quality as a result of extensive non-specific cross-hybridisation to non-globin probes reducing visualisation of non-globin transcripts. Therefore, the quality and accuracy of gene expression profiles obtained from peripheral blood is highly reliant on the effectiveness of globin reduction carried out prior to microarray probe generation. Technologies available to reduce globin mRNA have been shown to efficiently increase the sensitivity of transcript detection (Field et al. 2007; Li et al. 2008; Liu et al. 2006) but can also reduce the signal intensities achieved for some genes (Field et al. 2007). Importantly, methods of globin reduction have also been shown to introduce changes in the transcriptome profile observed (Feezor et al. 2004; Liu et al. 2006). A potential solution to the problem of globin reduction prior to gene expression profiling of whole blood has been launched by NuGEN Technologies^™^ (California, U.S.A.) in the form of the Ovation^™^ RNA amplification system V2. The Ovation system utilises a single primer, isothermal linear amplification (SPIA) method (Kurn et al. 2005) to generate single-strand cDNA microarray probes suitable for use with Affymetrix GeneChips^™^. Reproducibility studies and comparison to other microarray methods have illustrated a high degree of consistence and greater hybridisation specificity when exploiting sscDNA: DNA hybridisation compared to cRNA:DNA on microarrays (Barker et al. 2005). The NuGEN^™^ Ovation Whole Blood System does not require globin reduction strategies. Whilst globin transcripts are still converted to ssDNA, the high abundance of these do not present an issue in microarray hybridisations due to the increased specificity of sscDNA probes compared to cRNA probes. This system therefore has the potential to allow whole blood transcriptional profiling with decreased sample processing steps and circumvention of potential artefactual modulation of the transcriptome associated with globin reduction strategies. Ultimately, the utility of any whole blood profiling approach is dependent on its ability to provide a faithful representation of the individual hematopoietic lineage transcriptomes present in blood.

In the present study we therefore sought to identify a workflow which potentially allows (a) efficient and immediate stabilisation of collected peripheral whole blood and (b) microarray probe generation without the requirement for globin reduction. In addition to establishing the robustness and reproducibility of such methods, we also gauged the representation of specific hematopoietic lineage transcriptomes afforded by the evaluated approaches in order to identify an optimal workflow.

## Materials and Methods

### Sample collection and processing

**Figure 1:**
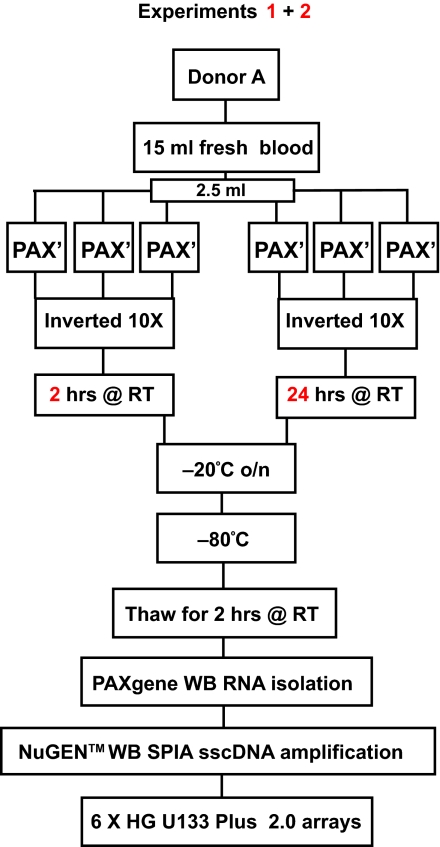
Experiment workflow utilised for storage and processing of PAXgene^™^-collected whole blood Peripheral whole blood was collected from a single donor on each of two separate days (Experiments 1 and 2). 2.5 ml whole blood was dispensed in each of 6 PAXgene^™^ tubes for each experiment. Following inversion (10 times), tubes were stored at room temperature for 2 hrs or 24 hrs. All samples were then transferred to −20 °C overnight followed by −80 °C for storage until RNA isolation procedures were performed using the PAXgene^™^ RNA isolation kit. sscDNA probes were synthesised by NuGEN Whole Blood SPIA amplification using the NuGEN Ovation^™^ RNA amplification V2 and Whole Blood reagent system. sscDNA was then hybridised to Affymetrix Human Genome U133 Plus 2.0 arrays. WB—Whole Blood, HG = Human Genome, PAX′ = PAXgene^™^ and RT = Room Temperature.

Blood samples were collected from an appropriately consented healthy donor using the PAXgene^™^ collection tube system (PreAnalytiX, Hornbrechtikon, Switzerland). Blood was drawn by standard phlebotomy procedures, with 2.5 ml dispensed into each of 6 PAXgene^™^ tubes and processed according to the manufacturer’s directions (Fig. 1). Briefly, half of the samples collected (n = 3) were maintained at room temperature for 2 hrs and the other half for 24 hrs, both within the manufacturers’ stated period of storage stability of 2–72 hrs. All samples were inverted 10 times and frozen at −20 °C overnight and then moved to −80 °C for storage until RNA isolation (Experiment 1). This same experimental procedure was repeated using a fresh blood draw obtained from the same donor on a separate day (Experiment 2).

Additional blood samples from the same subject were collected into K_3_-EDTA tubes and processed for full blood counts on a Sysmex XE 2100 fully automated full blood count analyser.

### Total RNA isolation

Frozen PAXgene^™^ tubes were thawed at room temperature for 2 hours followed by total RNA isolation according to the manufacturers’ instructions (PreAnalytiX, Hornbrechtikon, Switzerland). Briefly, PAXgene^™^ blood tubes were centrifuged for 10 minutes at 3000 × *g*, the supernatant removed and the cell pellet resuspended in 4 ml of RNase-free water. Samples were re-centrifuged for 10 minutes at 3000 × *g* and the supernatant removed. The cell pellet was resuspended in 350 μl Buffer BR1 and following the addition of 300 μl Buffer BR2 and 40 μl Proteinase K incubated for 10 minutes at 55 °C while shaking at 400 rpm. The resultant lysate was centrifuged through a PAXgene^™^ Shredder spin column at 16,300 × *g* for 3 minutes. The supernatant of the flow through was added to 350 μl of 100% ethanol, mixed and applied to a PAXgene^™^ RNA spin column and centrifuged for 1 minute at 16,300 × *g*. 350 μl of Buffer BR3 was added to the PAXgene^™^ RNA spin column and centrifuged for 1 minute at 16,300 × *g*. A DNase I incubation mix was prepared by combining 10 μl of DNase I and 70 μl of Buffer RDD per sample. DNase treatment was performed by pipetting 80 μl of DNase I incubation mix directly on to the PAXgene^™^ RNA spin column membrane and incubating at room temperature for 15 minutes. To wash the PAXgene^™^ RNA spin column 350 μl of Buffer BR3 was added and centrifuged for 1 minute at 16,300 × *g*. 500 μl of Buffer BR4 was added to the PAXgene^™^ RNA spin column and centrifuged for 1 minute at 16,300 × *g*. The addition of Buffer BR4 was repeated with a 3 minute 16,300 × *g* centrifugation followed by additional centrifugation for 1 minute at 16,300 × *g* to dry the column. RNA was eluted into a fresh 1.5 ml microcentrifuge tube using 40 μl of Buffer BR5 followed centrifugation for 1 minute at 16,300 × *g*. Elution was repeated with 40 μl of fresh Buffer BR5 giving a total elution volume of 80 μl. Eluted RNA was denatured for downstream applications by incubation at 65 °C for 5 minutes. Total RNA concentration was measured by Nanodrop ND-8000 (Nanodrop Technologies, Wilmington, DE, USE), and the integrity of the extracted total RNA was assessed using RNA Nano Chips on an Agilent 2100 Bioanalyzer (Agilent Technologies, Santa Clara, CA, U.S.A). Agilent Bioanalyzer 2100 electropherograms were analysed by the Biosizing software 2100 Expert Version B.02.03.SI307 (Agilent Technologies, 2100 Bioanalyzer) to generate RNA integrity numbers (RIN) as an RNA quality control metric.

### Hematopoietic cell lineages

Hematopoietic cell lineage transcript expression data was obtained from a number of sources (see Table 4). RNA derived in-house (B-cells, T-cells and Dendritic cells) for microarray hybridisation was obtained from frozen cell preparations purchased from Lonza Biosciences (Basel, Switzerland). Transcript expression data for a wide range of other cell lineages were obtained from public datasets deposited in the Gene Expression Omnibus (http://www.ncbi.nlm.nih.gov/geo/), accession numbers GSE1133 and GSE3982 (Jeffrey et al. 2006; Su et al. 2004). Taken together this dataset provided a source of mRNA expression data (derived from Affymetrix GeneChips^™^) from a wide range of hematopoietic cell lineages prepared by either density gradient, positive or negative paramagnetic bead isolation, and with or without stimulated differentiation *in vitro*.

### Microarray probe generation

Preparation of single stranded cDNA (sscDNA) SPIA probes for microarray hybridisation, without the requirement for globin reduction pretreatment, was carried out with 50 ng of total PAXgene^™^ RNA using NuGEN Technologies (California, U.S.A.) Ovation^™^ RNA amplification system V2 according to the manufacturers’ instructions. Synthesised sscDNA probe concentration was measured by Nanodrop ND-8000 (Nanodrop Technologies, Wilmington, DE, U.S.A). The NuGEN Technologies FL-Ovation^™^ cDNA Biotin Module V2 was used for sscDNA fragmentation and biotin labelling of 4.4 μg of the amplified sscDNA for subsequent hybridisation to the microarrays (Affymetrix, Santa Clara, CA, U.S.A). The quality of the sscDNA probes was assessed before and after fragmentation with the Agilent 2100 Bioanalyzer using RNA Nano chips (Agilent Technologies, Santa Clara, CA, U.S.A).

RNA (2 μg) prepared in-house from B-cells, T-cells and Dendritic cells was converted to cRNA, further processed according to Affymetrix (Santa Clara, CA, U.S.A) recommendations and quality assessed by Agilent 2100 Bioanalyser.

**Figure 2:**
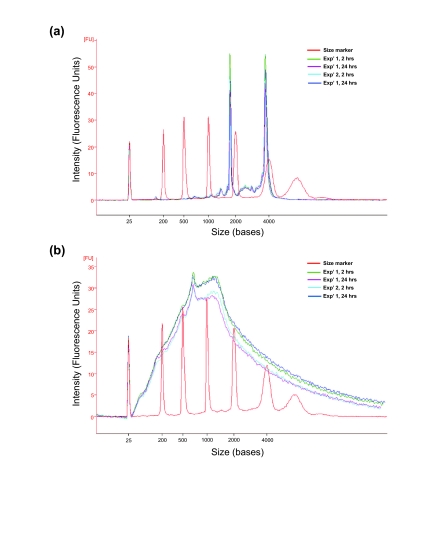
Bioanalyser electropherograms of total RNA isolated from PAXgene^™^ collected whole blood and subsequently synthesised sscDNA probes **(a)** Bioanalyzer electropherograms of a representative RNA from each experimental group are shown. **(b)** sscDNA quality assessed by distribution of size. The majority of sscDNA synthesised is in the order of approximately 1000 nucleotides. Bioanalyzer electropherograms of a representative sscDNA from each experimental group are shown. x-axis represents nucleotide length and y-axis is arbitrary signal intensity fluorescence units.

Nanodrop ND-8000 and Agilent 2100 Bioanalyzer were also utilised for concentration and quality assessment of sscDNA generated by NuGEN Ovation^™^ RNA amplification system V2 (Table 1, Fig. 2).

### Affymetrix GeneChips

For in-house prepared B-cell, T-cell and dendritic cell data, 15 μg of fragmented cRNA was hybridised to Affymetrix HG-U133A GeneChips, according to standard Affymetrix procedures. Data from GSE1133 and GSE3982 also resulted from hybridisation to HG-U133A GeneChips.

sscDNA probes generated from RNA isolated from PAXgene^™^ collected blood were hybridised to Affymetrix Human Genome U133 Plus 2.0 Arrays (Affymetrix, Santa Clara, CA, U.S.A.) as described in the Affymetrix Expression Analysis Technical Manual. Briefly, 4.4 μg of fragmented and labelled sscDNA, together with spiked hybridisation controls (GeneChip Expression 3′ Amplification Reagents—hybridisation controls), was hybridised for 18 hrs at 45 °C in a rotating oven. Following hybridisation GeneChip washing and staining was performed using the GeneChip Hybridisation Wash and Stain kit (Affymetrix, Santa Clara, CA, U.S.A.) on an Affymetrix GeneChip Fluidics Station 450 using the appropriate fluidic script for the U133 Plus 2.0 microarrays with sscDNA (FS450-0004). GeneChips were scanned immediately following staining in an Affymetrix GeneChip Scanner 3000 (Affymetrix, Santa Clara, U.S.A).

### Data analysis

Report files summarising the quality of target and control detection for each microarray were generated by GeneChip Operating Software Version 1.4 (GCOS) using the MAS5.0 algorithm (Affymetrix, Santa Clara, CA, U.S.A). All parameters (noise factor, background, and scaling factor) were acceptable by Affymetrix recommendations (Table 2).

Assessment of PAXgene^™^/NuGEN^™^ sscDNA whole blood data was performed using a combination of Affymetrix MAS5.0 feature extraction and RMA (Irizarry et al. 2003). Briefly, those probesets determined by MAS5.0 to be present in at least 25% of the PAXgene^™^ whole blood samples were selected for downstream analyses. Array files were subsequently processed by RMA, filtered on those MAS5.0-selected probesets. Data were Log_2_-transformed and those probesets with values greater than 6.5 in at least one sample selected for further analysis. Comparison between 2 hrs and 24 hrs PAXgene^™^ storage was performed by SAM analysis (Tusher et al. 2001) implemented in TM4 MEV (multi-experiment viewer) (Saeed et al. 2003) and using all samples, from both experiments 1 and 2 together, with a False Discovery Rate (FDR) of 0.05.

For assessment of any 3′ or 5′ bias of probeset signal intensity the chromosomal location of probe-sets were mapped from Affymetrix NetAffx annotation, allowing assessment of bias of signal intensity for those transcripts containing two or more probesets at distinct positions of the corresponding transcript.

To identify probesets most indicative of specific hematopoietic lineages, SAM analysis was performed with an FDR of 0 to select probesets significantly over-represented in one cell type compared to all other cell types within the same dataset. Following identification of probesets significantly over-represented in one particular lineage we applied a second filtering criterion of exclusion of those probesets with linear expression levels of less than 200 to maximise the likelihood of relevance of identified probesets being “diagnostic” of specific hematopoietic lineages.

CEL files for Paxgene^™^ data and internally generated hematopoietic lineage microarray data are available from Array Express (http://www.ebi.ac.uk/microarray-as/aer), accession number E-MEXP-1600.

## Results

### RNA yield and quality

In order to assess the yield and quality of the RNA obtained following PAXgene^™^ RNA stabilisation and isolation according to the workflow outlined in Figure 1, quality control analyses were carried out and yields are presented in Table 1. Analysis of total RNA quality by Agilent Bioanalyzer generated showed high RIN (RNA integrity numbers) for all samples (Table 1), revealing good quality RNA above the level traditionally deemed acceptable for microarray probe generation (RIN = 7.0) (Fig. 2, A). No significant differences in yield or quality of RNA obtained from samples collected (a) in different experiments or (b) as a result of PAXgene^™^ storage time were noted.

### Efficacy of sscDNA probe preparation from PAXgene^™^ whole blood RNA

All RNA samples were used to synthesize sscDNA probes for use in microarray gene expression profiling. Yields of sscDNA within the same experiments are tightly grouped and no significant difference between experiments or as a result of incubation times were observed (Table 1). sscDNA yields were consistently in excess of the 4.4 μg required for further processing for array hybridisations. Quality assessment by Agilent Bioanalyzer shows that the sscDNA fragments generated from whole blood RNA are of broad distribution in length, averaging approximately 1000 nucleotides, suggesting efficient sscDNA synthesis (Fig. 2, B). sscDNAs were fragmented and biotin-labelled in preparation for hybridisation and all resulted in fragments of ~50–200 nt (data not shown), similar to results previously reported using this method (Barker et al. 2005).

### Quality of microarray measurements

High quality control metrics for both RNA and sscDNA indicated successful sample preparation and all were subsequently hybridised to Affymetrix Human Genome U133 Plus 2.0 Arrays. All arrays within the study generated percentage present calls of between 60%–63%. Average background was 32.80 ± 0.31 (mean ± standard error), within the typical range of 20–100, the average noise factor was 1.43 ± 0.04 and the average scaling factor for all arrays was 1.56 ± 0.08. Finally, the β-actin 3′–5′ ratio were consistent between all arrays suggesting no significant degradation of samples. Neither experiment or incubation period of PAXgene^™^ whole blood contributed to any variation in quality control metrics (Table 2).

### Hematopoietic transcriptome stability in PAXgene^™^ Whole Blood samples

**Figure 3:**
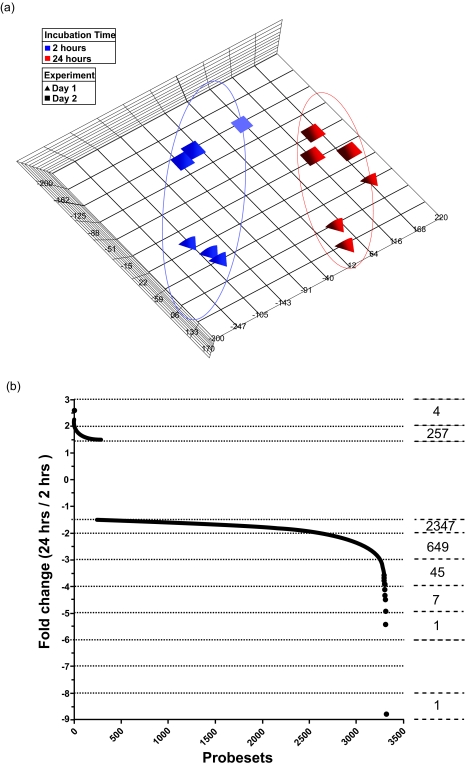
Analyses of array data derived from hybridisation of sscDNA probes generated from PAXgene^™^collected whole blood RNA **(a)** Principal Component Analysis of those probesets remaining after filtering for (i) present call by MAS5.0 in at least 25% of samples and (ii) signal intensity greater than 6.5 (Log_2_) in at least one sample following RMA processing of probesets derived from (i). Squares, experiment 1; triangles, experiment 2; blue, 2 hrs incubation; red, 24 hrs incubation. (**b**) Graphical representation of the number of probesets determined to be over- or under-represented in 24 hr compared to 2 hr stored samples following SAM analysis with an FDR of 0.05 and fold change ≥1.5. The number of probesets within each fold change range is indicated.

Following data normalisation, transformation and filtering, as outlined in materials and methods, Principal Component Analysis of remaining probesets was performed (Fig. 3a). As would be expected, distinct groupings of data according to experimental day were evident. Similarly, the PCA model revealed significant influence of PAXgene^™^ whole blood incubation period to variance within the dataset, for both experiments. SAM analysis was used with an FDR of 0.05 to identify those probesets differing in expression level in PAXgene^™^ whole blood samples stored at 24 hrs relative to 2 hrs. Those probesets fulfilling these criteria and which differ in expression level greater than 1.5-fold in 24 hr samples relative to 2 hrs are represented graphically in Figure 3b. This analysis identified a total of 3311 probesets differentially represented in PAXgene^™^ collected whole blood incubated at room temperature for 24 hrs compared to 2 hrs. The vast majority of these, 3050 (92.1%), represent probesets with lower signal intensities in 24 hr relative to 2 hr samples. Whilst a large amount of these (78.6%) are relatively modest 1.5 to 2-fold changes, a significant number are differentially represented at levels greater than 2-fold, with only 4 of these representing probesets over-represented in 24 hr samples. The complete annotated list of these probesets is available as Supplementary online Table 1.

We first considered the possibility whether those probesets under-represented in 24 hr samples were indicative of canonical degradation of mRNAs. To assess whether these could be due to 5′–3′ degradation of the mRNA transcripts we exploited the fact that several transcripts are represented by 2 or more probesets on the Affymetrix Human Genome U133 Plus 2.0 Arrays. The Affymetrix annotation file allows us to map the chromosomal location of these multiple probesets for the genes showing differential expression between 2 hrs and 24 hrs storage. This then allowed identification of which probeset is responsible for reduced signal intensity in the 24 hrs samples and subsequent assessment of whether this was due to a loss of the 3′ or 5′ probesets. Most genes were excluded from these analyses due to the presence of only one probeset or multiple probesets mapping to overlapping chromosomal regions. In total 117 transcripts were utilised to investigate a potential polarity effect (i.e. 3′ or 5′ degradation). Interestingly, of these 117 genes a clear degradation effect was observed with 94 genes displaying preferential loss of a 3′ probeset and only 23 genes showing loss of a 5′ probeset suggesting highly significant (P < 1 × 10^−7^, repeat random sampling) 3′ degradation.

### Faithfulness of hematopoietic cell lineage transcriptome representation in PAXgene^™^ whole blood expression profiles

It is essential that any workflow utilising whole blood provides a faithful representation of individual cell lineage transcriptomes. We wished to have an objective measurement of the probesets we had identified as “present” in our PAXgene^™^ analyses compared to probesets independently identified from samples where specific haematopoietic cell lineages had been isolated as being indicative of the identity of that particular lineage. We pursued this approach with the additional intention of investigating whether the marked under-representation of a large number of probesets following 24 hr PAXgene^™^ whole blood storage resulted from preferential reduction in signal intensity of probes accounted for by a particular hematopoietic lineage. ‘Signature probe-sets’ indicative for a number of individual haematopoietic cell lineages were estimated from both in-house and public data sets (Jeffrey et al. 2006; Su et al. 2004). These datasets were generated utilising a wide range of enrichment techniques; positive and/or negative selection and with or without *in vitro* stimulation and culture from either cord blood or peripheral blood (Table 4). Signature probesets were identified following SAM analysis with an FDR of 0, as indicated in materials and methods, from those found to be significantly over-represented in the cell-type of choice, compare to all other cell-types in that dataset. This data is summarised in Table 4 and the resultant annotated list of lineage-enriched probesets are available as Supplementary online Table 2.

To safeguard against any differences in hematopoietic cell lineages observed between our PAXgene dataset and others being due to abnormal levels of a particular cell lineage from the individual donor used in this study full haematopoietic analysis on blood samples from this individual was carried out and shown to be within healthy boundaries (Table 3).

**Figure 4:**
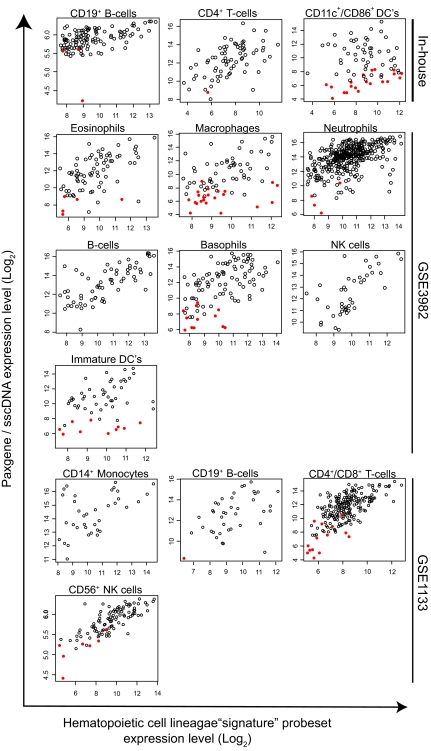
Scatterplots indicating the representation of lineage-enriched probesets in PAXgene^™^/sscDNA-derived data Log_2_-transformed expression data for those probesets indicative of specific lineages compared to expression data derived from PAXgene^™^/sscDNA hybridisations are indicated. Sources of each dataset are noted. Probesets passing the filters outlined in methods and materials but which were determined not to be expressed in the PAXgene^™^/sscDNA datasets are indicated as filled red circles. DCs, dendritic cells; NK, Natural killer cells.

Probesets determined to be hematopoietic cell lineage enriched remain well represented in the PAXgene^™^/sscDNA 2 hr/24 hr combined dataset (Table 4, Fig. 4), with several reaching 100% representation. Macrophages and dendritic cell transcript “signatures” appear most-under-represented in the PAXgene^™^/sscDNA whole blood expression data (71% and 75% respectively). Notably, those probesets determined to be sensitive to PAXgene^™^ whole blood incubation period do not appear to be contributed to by any particular individual lineage under study. Whilst the high degree of representation of individual hematopoietic lineage transcriptome in PAXgene^™^/sscDNA data was encouraging it was important to establish that this was not due solely to high abundance transcripts being interrogated. We therefore investigated the relationship between abundance of transcripts identified as being indicative of a particular lineage compared to transcript abundance observed in the PAXgene^™^/sscDNA data (Fig. 4). The small number of lineage enriched probesets, not represented in the PAXgene^™^/sscDNA dataset, appear over a range of abundances but are mostly lowly expressed in both the original and PAXgene^™^ datasets. Importantly, several low abundant genes within the isolated cell data sets are robustly detected in the PAXgene^™^ dataset (Fig. 4). Interestingly, there is a general concordance between the levels of expression in the original cell-line specific data and in our PAXgene^™^ whole blood data set.

There is only low representation (0%–2%) of any of these lineage specific probesets in those determined to be unstable as a result of storage times, therefore no loss of a particular cell lineage accounts for these changes.

## Discussion

The ability to robustly detect and measure biological variation associated with disease or pharmacologic intervention in whole blood clinical samples is key to the future discovery of surrogate disease biomarkers leading to translational research and personalised medicine. To this end we have assessed a workflow which allows microarray gene expression analysis of whole blood using PAXgene^™^ RNA stabilisation and subsequent sscDNA microarray probe generation without the requirement for globin reduction practices.

Despite claims that PAXgene^™^ technology efficiently stabilises whole blood samples for up to 3 days at room temperature, reports of reduced RNA quality with longer PAXgene^™^ storage and importantly, instability of a small number of studied transcripts despite retention of RNA integrity (Kagedal et al. 2005), within these recommended storage limits, warranted further investigation of transcriptome stability in PAXgene^™^ collected whole blood. To assess the stability of the entire whole blood transcriptome under storage conditions reflecting likely sample collection schema in clinical environments and within the recommended limits of the PAXgene^™^ technology we used Affymetrix GeneChip array technology and PAXgene^™^ collected whole blood stored at room temperature for either 2 or 24 hrs.

Our data demonstrate that PAXgene^™^ stabilised peripheral blood samples stored at room temperature for either 2 hrs or 24 hrs results in high quality RNA and generate ample sscDNA microarray probes for gene expression analysis, with subsequent arrays being of high quality with significantly high present calls (60%–62%), despite the omission of any globin interference reduction strategies. As would be expected, further analysis of array datasets revealed significant contribution of (a) experimental day and (b) incubation period to overall variance within the data. However, despite the preservation of RNA quality, storage for 24 hrs at room temperature resulted in significant transcriptome alterations compared to storage for 2 hrs, with the majority (92.1%) representing probesets under-represented in 24 hr samples at levels greater than 1.5-fold. We interpret this to indicate that intracellular RNA stabilisation is not 100% complete following 2 hrs storage at room temperature. Those transcripts for which we were able to further investigate polarity of any degradation effect substantiated this notion, with significantly lower signal intensity being attributed to the 3′ end of transcripts. Whilst this in agreement with incomplete RNA stabilisation it also indicates that this does not necessarily result in canonical RNA degradation, with this data indicating loss of poly(A) sequences, the effects of which would of course be expected to manifest most profoundly in procedures utilising poly(A) tails for reverse transcription. The clinical implication of these findings are significant as variation in sample storage or transportation time will lead to *ex vivo* transcriptome alterations which may mask any clinically relevant biological variation present within those transcripts affected by storage artefacts. However, it is important to note that Affymetrix expression array GeneChips are focussed to the 3′ end of transcripts and this should be considered when evaluating our data in context with competitor microarray platforms.

Whilst size, nucleotide composition, secondary structure properties and abundance of transcripts could all conceivably account in part for the storage time dependent transcriptome alterations noted here we also considered it possible, albeit unlikely after more than 2 hours, that this could indicate differential lysis of a particular hematopoietic lineage within the PAXgene^™^ lysis matrix, thereby fostering RNA degradation within those cells. Notably, signal intensities of the vast majority (74%) of those transcripts identified as being PAXgene^™^ storage period sensitive at 24 hrs are within the upper 50% of signal intensity distribution for those probesets obtained at 2 hrs (data not shown), indicating that increased periods of whole blood storage in the PAXgene^™^ system predominantly affects transcripts of highest abundance.

Comparison of our PAXgene^™^ sample data set with expression data of transcripts determined to be indicative of specific lineage populations, we demonstrated that the 24 hr under-represented transcripts appeared to be cell-lineage independent. Similarly, pathway mapping analysis did not reveal any obvious networks that could be attributed to cell-specific biological functions (data not shown). The underlying factors influencing transcript specific degradation and significant storage time dependent under-representation within the PAXgene^™^ samples are therefore undetermined at this time.

Our data are concordant with several reports that RNA integrity remains intact following extended room temperature storage times following PAXgene^™^ blood sample collection (Chai et al. 2005; Thach et al. 2003; Wang et al. 2004). However, we have shown degradation and significant under-representation of a large number of transcripts following just 24 hrs room temperature storage, supporting previous reports (Kagedal et al. 2005) of sample instability, but contrary to others showing no change in transcript levels up to 5 days of room temperature storage (Chai et al. 2005). It is likely that this latter discrepancy is due to the use of qPCR methods to analyse only a small number of specific transcripts (Chai et al. 2005; Thach et al. 2003; Wang et al. 2004), rather than global profiling approaches. Reports have also compared longer storage times (24 hrs and 5 days) demonstrating no alterations in transcript representation (Chai et al. 2005). However, with the omission of shorter time points interpretation of whether the abundance of these transcripts has already altered from their original basal levels at time of collection is not possible.

We report the generation of high quality global gene expression data from PAXgene^™^ peripheral blood samples, which we believe will be vital for future biomarker discovery and interrogation within clinical samples. However, considering transcriptome alterations observed within the storage time range recommended by the manufacturers it will be imperative to instigate a fixed storage time for all clinical samples intended for direct comparison within a given study.

A further aim of this study was to assess the suitability of a single-primer isothermal linear amplification (SPIA) method for application with Affymetrix GeneChip microarrays for whole blood transcriptome analysis potentially mitigating the need for globin reduction. Globin reduction practices in whole blood RNA sample processing have previously been shown to alter global gene expression profiles obtained (Feezor et al. 2004; Liu et al. 2006) and significantly increase sample handling. Using this SPIA technology we synthesised high quality sscDNA in ample quantity from PAXgene^™^ samples to carry out hybridisation to microarrays. sscDNA probes for microarrays have previously been shown to provide high specificity of hybridisation and consequently provide increased percentage present calls, as a result of reduced cross-hybridisation to mismatch control probes, and with this workflow we achieved a minimum of 60 percent present calls on Affymetrix Genechips. Therefore, we have demonstrated that NuGEN Technologies Whole Blood Ovation^™^ RNA amplification for probe generation permits analysis of gene expression profiles from whole blood with minimal handling steps without the requirement for potentially transcriptome-modifying globin reduction practices that were previously required and recommended by the Best Practices Blood Handling for Array Based Expression Working Group (www.affymetrix.com/community/standards/blood_protocol.affx). Importantly, the gene expression data sets obtained as a result of this workflow result in excellent representation of individual hematopoietic cell lineages with a wide spectrum of transcript abundances, thereby supporting the notion that efficient whole blood expression profiling studies can be achieved without requirement for lineage or cell population enrichment strategies and without loss of data contributed by specific lineages. Whilst the high levels of representation of transcripts indicative of specific cell lineages in the PAXgene^™^/sscDNA dataset was remarkable, as expected several of these did not reach 100% and we consider this a likely reflection of the methods employed for individual lineage enrichment. Notably, those lineages with lowest representation (approximately 70%) result from enrichment schemes which include subsequent *in vitro* stimulation and culture and with transcriptome profiles likely least reflective of those lineages *in vivo*.

We conclude that robust global gene expression profiling from whole blood without the requirement for globin reduction processing schemes and with maximal representation of individual hematopoietic lineages can be achieved by combination of PAXgene^™^ RNA stabilisation procedures, albeit with careful consideration to fixed incubation periods for all samples within a clinical collection, together with NuGEN Technologies Whole Blood Ovation^™^ RNA amplification system for microarray probe generation.

## Figures and Tables

**Table 1:** Yield and quality metrics for isolated total RNA and synthesized single stranded cDNA probes.

Experiment	Storage time (hours)	RNA yield (μg/ml)	Bioanalyser RIN	sscDNA yield (μg)
**1**	**2**	4.87 ± (0.18)	9.0 ± (0.06)	9.3 ± (0.43)
**1**	**24**	5.94 ± (0.24)	8.5 ± (0.07)	8.9 ± (0.27)
**2**	**2**	5.86 ± (0.31)	9.3 ± (0.03)	8.1 ± (0.25)
**2**	**24**	4.55 ± (0.61)	8.8 ± (0.03)	7.7 ± (0.10)

Mean quality metrics for total RNA isolated from 2 hrs (n = 3) and 24 hrs (n = 3) stored PAXgene^™^ whole blood samples from both experiments 1 and 2 are indicated. Numbers in parenthesis represent s.e.m. Storage time refers to the period the PAXgene^™^ collected whole blood samples were stored at room temperature prior to storage at −20 °C. Room temperature incubations are a model of the period samples spend in the clinic or in transit before processing by the appropriate laboratory. RNA quality metrics were obtained by Nanodrop ND-8000 spectrophotometery and by Agilent 2100 Bioanalyzer. RIN; RNA Integrity Number.

**Table 2:** Quality metrics following hybridisation of sscDNA probes generated from whole blood RNA to Affymetrix Human Genome U133 Plus 2.0 Arrays.

Experiment	Storage time (hours)	% Present	Mean% present	Mean actin 3′5′ ratio	Mean GAPDH 3′5′ ratio
1	2	62.5 60.8 63	62.1 + (0.64)	2.06 + (0.04)	1.59 + (0.01)
1	24	62.6 63.8 61.7	62.7 + (0.63)	2.23 + (0.08)	1.51 + (0.03)
2	2	62.5 62.9 61.4	62.3 + (0.44)	2.47 + (0.13)	1.67 + (0.04)
2	24	61.8 60.3 62.4	61.5 + (0.63)	2.52 + (0.20)	1.63 + (0.07)

Mean quality metrics for microarrays from hybridisation of sscDNA generated from PAXgene^™^ whole blood RNA stored for 2 hrs (n = 3) and 24 hrs (n = 3). PAXgene^™^ whole blood samples from both experiments 1 and 2 are indicated. Numbers in parenthesis represent s.e.m. Quality metrics reflect GCOS report data generated following MAS5.0 feature extraction.

**Table 3:** Donor hematological full blood counts.

Cell type	Donor A
RBC (× 10^12^/L)	5.3 (4.5–6.0)
WBC (× 10^9^/L)	6.3 (4.0–11.0)
Neutrophils (× 10^9^/L)	3.4 (2.0–7.5)
Lymphocytes (× 10^9^/L)	2.2 (1.5–4.0)
Monocytes (× 10^9^/L)	0.4 (0.2–0.8)
Eosinophils (× 10^9^/L)	0.25 (0–0.4)
Basophils (× 10^9^/L)	0 (0–0.1)
Platelets (× 10^9^/L)	198 (150–400)

Full haematological analysis of whole blood samples illustrate that all haematopoietic cell lineages are present in concentrations within the normal range in donor A. RBC = Red blood cells, WBC = White blood cells. Parenthesis = ref. range for males.

**Table 4:** Source details of hematopoietic cell lineages.

Cell type	Dataset	Purity	Markers	Enrichment	Positive selection antibody	Origin
CD19^+^ B-Cells	In-house	>85%	CD19	Negative selection	n/a	Cord blood
CD4^+^ T-Cells	In-house	>90%	CD4	Negative selection	n/a	Peripheral blood
Dendritic Cells	In-house	>85%	CD14, CD56	Leukopheresis and density gradient. *In vitro* differentiation.	n/a	Peripheral blood
Eosinophils	GSE3982	97%	CD16	Percoll gradient	n/a	Peripheral blood
Macrophages	GSE3982	Unknown	Unknown	Ficoll gradient, positive selection, GM-CSF	Anti-CD14	Peripheral blood
Neutrophils	GSE3982	>97%	CD16, CD62L	Ficoll gradient, negative selection	n/a	Peripheral blood
B-Cells	GSE3982	>96%	CD19	Ficoll gradient, positive selection	Anti-CD19	Peripheral blood
Basophils	GSE3982	>96%	CCR3	Ficoll gradient, negative selection followed by positive selection	Anti-CCR3	Peripheral blood
NK Cells	GSE3982	>96%	CD16, CD56	Ficoll gradient, positive selection	Anti-CD16/CD56	Peripheral blood
Immature Dendritic Cells	GSE3982	Unknown	Unknown	Ficoll gradient, positive selection, IL14/GM-CSF differentiation	Anti-CD14	Peripheral blood
CD14^+^ Monocytes	GSE1133	Unknown	CD14	Density gradient and positive selection	Anti-CD14	Peripheral blood
CD19^+^ B-Cells	GSE1133	Unknown	CD19	Negative selection	n/a	Peripheral blood
CD4^+^/CD8^+^ T-Cells	GSE1133	Unknown	CD4/CD8	Unknown	n/a	Peripheral blood
CD56^+^ NK Cells	GSE1133	>90%	CD16, CD56	Unknown	n/a	Peripheral blood

Preparation details for datasets utilised for generation of probesets indicative of specific hematopoietic cell lineages are shown for both in-house generated data and GSE3982 and GSE1133. Prepared cells are from a variety of sources and derived by a number of methods, including positive and negative selection (or both) and with or without *in vitro* differentiation procedures.

**Table 5:** Representation of probesets indicative of specific hematopoietic cell lineages in the PAXgene^™^ whole blood/sscDNA dataset.

Cell type	Dataset	Number of probesets indicative of cell lineage	Number of cell-lineage “signature” probesets present in PAXgene^™^/sscDNA dataset	Representation of cell-lineage “signature” probesets present in PAXgene^™^/sscDNA dataset (%)	Cell lineage “signature” probesets also identified to be storage time-sensitive (%)
CD19^+^ B-Cells	In-house	119	116	97	2.0
CD4^+^ T-Cells	In-house	34	34	100	3.0
Dendritic Cells	In-house	52	39	75	2.0
Eosinophils	GSE3982	78	73	94	8.2
Macrophages	GSE3982	76	54	71	3.7
Neutrophils	GSE3982	365	361	99	4.1
B-Cells	GSE3982	62	62	100	4.8
Basophils	GSE3982	102	90	88	8.9
NK Cells	GSE3982	41	41	100	0
Immature Dendritic Cells	GSE3982	56	46	82	0
CD14^+^ Monocytes	GSE1133	35	35	100	2.9
CD19^+^ B-Cells	GSE1133	39	38	97	5.3
CD4^+^/CD8^+^ T-Cells	GSE1133	224	209	94	3.4
CD56^+^ NK Cells	GSE1133	106	99	93	4.0

The number of probesets determined, as outlined in the materials and methods section, to be most indicative of specific hematopoietic cell lineages from in-house generated array data and from GSE3982 (Jeffrey et al. 2006) and GSE1133 (Su et al. 2004) are indicated. The number or proportion of these present in processed and filtered data derived from hybridisation of sscDNA probes derived from RNA isolated from PAXgene^™^ collected whole blood are indicated. The number of those probesets for each lineage which were also identified to be incubation period sensitive is also presented. NK cells; Natural Killer Cells.

## References

[b1-bmi-03-403] Alizadeh AA Eisen MB Davis RE Ma C Lossos IS Rosenwald A Boldrick JC Sabet H Tran T Yu X Powell JI Yang L Marti GE Moore T Hudson J JR Lu L Lewis DB Tibshirani R Sherlock G Chan WC Greiner TC Weisenburger DD Armitage JO Warnke R Levy R Wilson W Grever MR Byrd JC Botstein D Brown PO Staudt LM 2000 Distinct types of diffuse large B.-cell lymphoma identified by gene expression profiling Nature 403 503 11 10676951 10.1038/35000501

[b2-bmi-03-403] Baechler EC Batliwalla FM Karypis G Gaffney PM Ortmann WA Espe KJ Shark KB Grande WJ Hughes KM Kapur V Gregersen PK Behrens TW 2003 Interferon-inducible gene expression signature in peripheral blood cells of patients with severe lupus. Proc. Natl. Acad. Sci. U.S.A 100 2610 5 12604793 10.1073/pnas.0337679100 PMC151388

[b3-bmi-03-403] Barker CS Griffin C Dolganov GM Hanspers K Yang JY Erle DJ 2005 Increased DNA microarray hybridization specificity using sscDNA targets BMC Genomics 6 57 15847692 10.1186/1471-2164-6-57 PMC1090574

[b4-bmi-03-403] Bennett L Palucka AK Arce E Cantrell V Borvak J Banchereau J Pascual V 2003 Interferon and granulopoiesis signatures in systemic lupus erythematosus blood. J. Exp. Med 197 711 23 12642603 10.1084/jem.20021553 PMC2193846

[b5-bmi-03-403] Borovecki F Lovrecic L Zhou J Jeong H Then F Rosas HD Hersch SM Hogarth P Bouzou B Jensen RV Krainc D 2005 Genomewide expression profiling of human blood reveals biomarkers for Huntington’s disease. Proc. Natl. Acad. Sci. U.S.A 102 11023 8 16043692 10.1073/pnas.0504921102 PMC1182457

[b6-bmi-03-403] Bullinger L Dohner K Bair E Frohling S Schlenk RF Tibshirani R Dohner H Pollack JR 2004 Use of gene-expression profiling to identify prognostic subclasses in adult acute myeloid leukemia. N. Engl. J. Med 350 1605 16 15084693 10.1056/NEJMoa031046

[b7-bmi-03-403] Burczynski ME Dorner AJ 2006 Transcriptional profiling of peripheral blood cells in clinical pharmacogenomic studies Pharmacogenomics 7 187 202 16515398 10.2217/14622416.7.2.187

[b8-bmi-03-403] Burczynski ME Peterson RL Twine NC Zuberek KA Brodeur BJ Casciotti L Maganti V Reddy PS Strahs A Immermann F Spinelli W Schwertschlag U Slager AM Cotreau MM Dorner AJ 2006 Molecular classification of Crohn’s disease and ulcerative colitis patients using transcriptional profiles in peripheral blood mononuclear cells. J. Mol. Diagn 8 51 61 16436634 10.2353/jmoldx.2006.050079 PMC1867573

[b9-bmi-03-403] Burczynski ME Twine NC Dukart G Marshall B Hidalgo M Stadler WM Logan T Dutcher J Hudes G Trepicchio WL Strahs A Immermann F Slonim DK Dorner AJ 2005 Transcriptional profiles in peripheral blood mononuclear cells prognostic of clinical outcomes in patients with advanced renal cell carcinoma. Clin. Cancer Res 11 1181 9 15709187

[b10-bmi-03-403] Chai V Vassilakos A Lee Y Wright JA Young AH 2005 Optimization of the PAXgene blood RNA extraction system for gene expression analysis of clinical samples. J. Clin. Lab Anal 19 182 8 16170815 10.1002/jcla.20075 PMC6807908

[b11-bmi-03-403] Crow MK Wohlgemuth J 2003 Microarray analysis of gene expression in lupus. Arthritis Res. Ther 5 279 87 14680503 10.1186/ar1015 PMC333417

[b12-bmi-03-403] Debey S Zander T Brors B Popov A Eils R Schultze JL 2006 A highly standardized, robust, and cost-effective method for genomewide transcriptome analysis of peripheral blood applicable to large-scale clinical trials Genomics 87 653 64 16387473 10.1016/j.ygeno.2005.11.010

[b13-bmi-03-403] Dumeaux V Lund E Borresen-Dale A 2008 Comparison of globin RNA processing methods for genomewide transcriptome analysis from whole blood Biomarkers in Medicine 2 11 21 10.2217/17520363.2.1.11 20477359

[b14-bmi-03-403] Feezor RJ Baker HV Mindrinos M Hayden D Tannahill CL Brownstein BH Fay A Macmillan S Laramie J Xiao W Moldawer LL Cobb JP Laudanski K Miller-Graziano CL Maier RV Schoenfeld D Davis RW Tompkins RG 2004 Whole blood and leukocyte RNA isolation for gene expression analyses Physiol. Genomics 19 247 54 15548831 10.1152/physiolgenomics.00020.2004

[b15-bmi-03-403] Field LA Jordan RM Hadix JA Dunn MA Shriver CD Ellsworth RE Ellsworth DL 2007 Functional identity of genes detectable in expression profiling assays following globin mRNA reduction of peripheral blood samples. Clin. Biochem 40 499 502 17303101 10.1016/j.clinbiochem.2007.01.004

[b16-bmi-03-403] Golub TR Slonim DK Tamayo P Huard C Gaasenbeek M Mesirov JP Coller H Loh ML Downing JR Caligiuri MA Bloomfield CD Lander ES 1999 Molecular classification of cancer: class discovery and class prediction by gene expression monitoring Science 286 531 7 10521349 10.1126/science.286.5439.531

[b17-bmi-03-403] Han GM Chen SL Shen N Ye S Bao CD Gu YY 2003 Analysis of gene expression profiles in human systemic lupus erythematosus using oligonucleotide microarray. Genes Immun 4 177 86 12700592 10.1038/sj.gene.6363966

[b18-bmi-03-403] Hershey AD Tang Y Powers SW Kabbouche MA Gilbert DL Glauser TA Sharp FR 2004 Genomic abnormalities in patients with migraine and chronic migraine: preliminary blood gene expression suggests platelet abnormalities Headache 44 994 1004 15546262 10.1111/j.1526-4610.2004.04193.x

[b19-bmi-03-403] Irizarry RA Hobbs B Collin F Beazer-Barclay YD Antonellis KJ Scherf U Speed TP 2003 Exploration, normalization, and summaries of high density oligonucleotide array probe level data Biostatistics 4 249 64 12925520 10.1093/biostatistics/4.2.249

[b20-bmi-03-403] Jeffrey KL Brummer T Rolph MS Liu SM Callejas NA Grumont RJ Gillieron C Mackay F Grey S Camps M Rommel C Gerondakis SD Mackay CR 2006 Positive regulation of immune cell function and inflammatory responses by phosphatase PAC-1. Nat. Immunol 7 274 83 16474395 10.1038/ni1310

[b21-bmi-03-403] Kagedal B Lindqvist M Farneback M Lenner L Peterson C 2005 Failure of the PAXgene Blood RNA System to maintain mRNA stability in whole blood. Clin. Chem. Lab Med 43 1190 2 16232084 10.1515/CCLM.2005.206

[b22-bmi-03-403] Kurn N Chen P Heath JD Kopf-Sill A Stephens KM Wang S 2005 Novel isothermal, linear nucleic acid amplification systems for highly multiplexed applications. Clin. Chem 51 1973 81 16123149 10.1373/clinchem.2005.053694

[b23-bmi-03-403] Li L Ying L Naesens M Xiao W Sigdel T Hsieh S Martin J Chen R Liu K Mindrinos M Davis R Sarwal M 2008 Interference of globin genes with biomarker discovery for allograft rejection in peripheral blood samples Physiol. Genomics 32 190 7 17971501 10.1152/physiolgenomics.00216.2007

[b24-bmi-03-403] Liu J Walter E Stenger D Thach D 2006 Effects of globin mRNA reduction methods on gene expression profiles from whole blood. J. Mol. Diagn 8 551 8 17065423 10.2353/jmoldx.2006.060021 PMC1876175

[b25-bmi-03-403] Maas K Chan S Parker J Slater A Moore J Olsen N Aune TM 2002 Cutting edge: molecular portrait of human autoimmune disease. J. Immunol 169 5 9 12077221 10.4049/jimmunol.169.1.5

[b26-bmi-03-403] Mclean LA Gathmann I Capdeville R Polymeropoulos MH Dressman M 2004 Pharmacogenomic analysis of cytogenetic response in chronic myeloid leukemia patients treated with imatinib. Clin. Cancer Res 10 155 65 14734464 10.1158/1078-0432.ccr-0784-3

[b27-bmi-03-403] Mohr S Liew CC 2007 The peripheral-blood transcriptome: new insights into disease and risk assessment. Trends Mol. Med 13 422 32 17919976 10.1016/j.molmed.2007.08.003

[b28-bmi-03-403] Moore DF Li H Jeffries N Wright V Cooper RA Jr Elkahloun A Gelderman MP Zudaire E Blevins G YU H Goldin E Baird AE 2005 Using peripheral blood mononuclear cells to determine a gene expression profile of acute ischemic stroke: a pilot investigation Circulation 111 212 21 15630028 10.1161/01.CIR.0000152105.79665.C6

[b29-bmi-03-403] Moos PJ Raetz EA Carlson MA Szabo A Smith FE Willman C Wei Q Hunger SP Carroll WL 2002 Identification of gene expression profiles that segregate patients with childhood leukemia. Clin. Cancer Res 8 3118 30 12374679

[b30-bmi-03-403] Muller MC Merx K Weisser A Kreil S Lahaye T Hehlmann R Hochhaus A 2002 Improvement of molecular monitoring of residual disease in leukemias by bedside RNA stabilization Leukemia 16 2395 9 12454744 10.1038/sj.leu.2402734

[b31-bmi-03-403] Nowicki MO Pawlowski P Fischer T Hess G Pawlowski T Skorski T 2003 Chronic myelogenous leukemia molecular signature Oncogene 22 3952 63 12813469 10.1038/sj.onc.1206620

[b32-bmi-03-403] Pahl A Brune K 2002 Stabilization of gene expression profiles in blood after phlebotomy. Clin. Chem 48 2251 3 12446485

[b33-bmi-03-403] Rainen L Oelmueller U Jurgensen S Wyrich R Ballas C Schram J Herdman C Bankaitis-Davis D Nicholls N Trollinger D Tryon V 2002 Stabilization of mRNA expression in whole blood samples. Clin. Chem 48 1883 90 12406972

[b34-bmi-03-403] Saeed AI Sharov V White J Li J Liang W Bhagabati N Braisted J Klapa M Currier T Thiagarajan M Sturn A Snuffin M Rezantsev A Popov D Ryltsov A Kostukovich E Borisovsky I Liu Z Vinsavich A Trush V Quackenbush J 2003 TM4: a free, open-source system for microarray data management and analysis Biotechniques 34 374 8 12613259 10.2144/03342mt01

[b35-bmi-03-403] Su AI Wiltshire T Batalov S Lapp H Ching KA Block D Zhang J Soden R Hayakawa M Kreiman G Cooke MP Walker JR Hogenesch JB 2004 A gene atlas of the mouse and human protein-encoding transcriptomes. Proc. Natl. Acad. Sci. U.S.A 101 6062 7 15075390 10.1073/pnas.0400782101 PMC395923

[b36-bmi-03-403] Tang Y Gilbert DL Glauser TA Hershey AD Sharp FR 2005 Blood gene expression profiling of neurologic diseases: a pilot microarray study. Arch. Neurol 62 210 5 15710849 10.1001/archneur.62.2.210

[b37-bmi-03-403] Thach DC Lin B Walter E Kruzelock R Rowley RK Tibbetts C Stenger DA 2003 Assessment of two methods for handling blood in collection tubes with RNA stabilizing agent for surveillance of gene expression profiles with high density microarrays J. Immunol. Methods 283 269 79 14659918 10.1016/j.jim.2003.10.004

[b38-bmi-03-403] Thorn I Olsson-Stromberg U Ohlsen C Jonsson AM Klangby U Simonsson B Barbany G 2005 The impact of RNA stabilization on minimal residual disease assessment in chronic myeloid leukemia Haematologica 90 1471 6 16266893

[b39-bmi-03-403] Tusher VG Tibshirani R Chu G 2001 Significance analysis of microarrays applied to the ionizing radiation response. Proc. Natl. Acad. Sci. U.S.A 98 5116 21 11309499 10.1073/pnas.091062498 PMC33173

[b40-bmi-03-403] Twine NC Stover JA Marshall B Dukart G Hidalgo M Stadler W Logan T Dutcher J Hudes G Dorner AJ Slonim DK Trepicchio WL Burczynski ME 2003 Disease-associated expression profiles in peripheral blood mononuclear cells from patients with advanced renal cell carcinoma. Cancer Res 63 6069 75 14522937

[b41-bmi-03-403] Valk PJ Verhaak RG Beijen MA Erpelinck CA Barjesteh Van Waalwijk VAN Doorn-Khosrovani S Boer JM Beverloo HB Moorhouse MJ Van Der Spek PJ Lowenberg B Delwel R 2004 Prognostically useful gene-expression profiles in acute myeloid leukemia. N. Engl. J. Med 350 1617 28 15084694 10.1056/NEJMoa040465

[b42-bmi-03-403] Wang J Robinson JF Khan HM Carter DE Mckinney J Miskie BA Hegele RA 2004 Optimizing RNA extraction yield from whole blood for microarray gene expression analysis. Clin. Biochem 37 741 4 15329310 10.1016/j.clinbiochem.2004.03.013

[b43-bmi-03-403] Yu SY Hu YW Liu XY Xiong W Zhou ZT Yuan ZH 2005 Gene expression profiles in peripheral blood mononuclear cells of SARS patients. World J. Gastroenterol 11 5037 43 16124062 10.3748/wjg.v11.i32.5037 PMC4321926

